# Donafenib suppresses glioblastoma progression: potential enhancement of therapeutic response in TMZ-resistant models

**DOI:** 10.1007/s00210-026-05631-z

**Published:** 2026-07-06

**Authors:** Emine Yazici, Aleyna Gezen, Merzuka Kalay, Orhan Kocak, Ece Oylumlu, Ece Simsek, Gamze Tanriover

**Affiliations:** 1 Department of Histology and Embryology, Faculty of Medicine, Akdeniz University, Antalya, Türkiye; 2 Department of Biology, Institute of Sciences, Akdeniz University, Antalya, Türkiye; 3 Department of Nutrition and Dietetics, Institute of Health Sciences, Akdeniz University, Antalya, Türkiye; 4 Department of Medical Biotechnology, Institute of Health Sciences, Akdeniz University, Antalya, Türkiye

**Keywords:** Glioblastoma, Temozolomide, Donafenib, P-ERK, Multikinase inhibitor

## Abstract

Glioblastoma (GBM) is the most aggressive primary brain tumor of the central nervous system and remains largely incurable due to the development of resistance to temozolomide (TMZ). Aberrant activation of growth factor–mediated signaling pathways, including VEGFR and MET, plays a critical role in GBM progression, highlighting the need for alternative therapeutic strategies. Donafenib (DNF) is a novel multikinase inhibitor targeting VEGFR, PDGFR, and RAF signaling pathways. In this study, we evaluated the effects of DNF on proliferation-associated signaling pathways in U87MG and T98G GBM cell lines. DNF treatment significantly reduced cell viability, colony formation, migration, and invasion. Moreover, immunocytochemical analyses revealed lower levels of immunoreactivity for several markers associated with tumor progression, including p-ERK, NF-κB p65, p-mTOR, p-MET, p-STAT3, and p-VEGFR, following DNF treatment. While PAMPA analysis indicated that DNF exhibits lower blood–brain barrier (BBB) permeability than TMZ, in silico modeling revealed that a substantial proportion of its metabolites may possess BBB-penetrating potential. Collectively, these findings suggest that DNF exerts antitumor effects in both TMZ-sensitive and TMZ-resistant GBM models and highlight its potential as a promising therapeutic candidate for further investigation.

**Supplementary Information:**

The online version contains supplementary material available at https://doi.org/10.1007/s00210-026-05631-z.

##  Introduction

Glioblastoma (GBM), with an incidence rate of 3.19 per 100,000 individuals annually, is the most common primary brain tumor observed in adults (Hansen et al. 2023). These tumors are characterized by rapid cellular proliferation and extensive invasiveness, although distant metastasis is rare. Despite significant advances in surgical and pharmacological treatments, GBM remains associated with a poor prognosis (DeCordova et al. 2020).

Due to the limited life expectancy of patients, the first clinical target is to enhance disease-free and progression-free survival. The treatment of GBM patients includes surgical resection followed by concurrent radiotherapy and chemotherapy (Shergalis et al. 2018). Temozolomide (TMZ) is a DNA alkylating chemotherapeutic used to treat GBM after its first FDA approval in 2005 (Stupp et al. 2005). Due to the widespread exposure of patients to TMZ and the mutation-prone structure of GBM, these tumors frequently develop resistance to TMZ (Jiapaer et al. 2018; Singh et al. 2021).

Targeted therapies, including small-molecule multikinase inhibitors have been demonstrated to improve survival rates in various cancers (Seebacher et al. 2019). Donafenib (DNF) is a novel multikinase inhibitor that targets key oncogenic pathways, including vascular endothelial growth factor receptor (VEGFR), platelet-derived growth factor receptor (PDGFR), and RAF kinases. It has also been shown to suppress tumor cell proliferation and angiogenesis (Qin et al. 2021). DNF is known to improve overall survival in these patients and clinical trials are ongoing in colorectal, thyroid and nasopharyngeal cancers (Keam and Duggan 2021; Qin et al. 2021).

Although DNF has demonstrated clinical benefits in several cancer types, including hepatocellular carcinoma, its role in GBM remains largely unexplored. Given the critical role of dysregulated signaling pathways in GBM progression and therapy resistance, investigating the effects of DNF in both TMZ-sensitive and TMZ-resistant GBM models is of particular importance.

In this study, we aimed to evaluate the effects of DNF on cell viability, tumor progression-related phenotypes, and key signaling pathways in GBM cell lines, and to explore its potential as a therapeutic agent in the context of TMZ resistance.

## Materials and methods

### Prediction of DNF metabolites using GLORYx

The three-dimensional (3D) structures of TMZ and DNF were obtained from the PubChem database (CID: 44,247,727). The Simplified Molecular Input Line Entry System (SMILES) notation of DNF was used as input for GLORYx, an in silico tool that predicts human-relevant metabolites by simulating phase I and phase II metabolic transformations (De Bruyn Kops et al. 2021). The resulting predicted metabolites were collected for further analysis.

### BBB permeability evaluation using LogBB_Pred

The BBB permeability of DNF and its predicted metabolites was assessed using the LogBB_Pred web server (Shaker et al. 2023). This platform is based on a Light Gradient Boosting Machine (LightGBM) algorithm for predictive modeling (Shaker et al. 2021). Each compound was individually analyzed using its SMILES representation. Compounds were classified as BBB-permeable (BBB +) or non-permeable (BBB −) based on the model output. Predicted LogBB values were also recorded to enable comparative analysis between the parent compound and its metabolites.

### Cell lines and culture conditions

The human GBM cell lines used in this study were U87MG and T98G. The U87MG and T98G cell lines were selected based on their well-established differential sensitivity to TMZ reported in the literature, where U87MG is commonly described as relatively TMZ-sensitive and T98G as relatively TMZ-resistant (Kochanowski et al. 2021). Both cell lines were maintained in Dulbecco’s Modified Eagle Medium (DMEM; Thermo Fisher Scientific, #11,965,092, Waltham, MA, USA) supplemented with 10% fetal bovine serum (FBS; Thermo Fisher Scientific, #A5256701), 2% L-glutamine, and 1% penicillin–streptomycin (P/S; Thermo Fisher Scientific, #15,140,122). Cells were cultured at 37 °C in a humidified incubator containing 5% CO₂ under standard cell culture conditions (Oylumlu et al. 2026). Upon reaching approximately 70% confluency, adherent cells were detached using 0.25% trypsin–EDTA solution (Thermo Fisher Scientific, #25,200,056) and passaged according to ATCC-recommended protocols.

### Cell viability assay

The effective concentrations of TMZ (Sigma-Aldrich, #T2577, USA) and DNF (Selleck Chemicals, #S9621, USA) in GBM cells were identified using an MTT (3-(4,5-Dimethylthiazol-2-yl)−2,5-Diphenyltetrazolium Bromide) Cell Proliferation Kit (Roche, #11,465,007,001, Basel, Switzerland). Based on the literature, TMZ was administered to experimental groups at doses ranging from 3 to 800 µM (Lee 2016). DNF was administered at concentrations ranging from 40 to 0.315 µM. At the conclusion of incubation, cell viability was evaluated according to the standard MTT assay protocol (Ülkeryıldız-Balçık et al. 2025; Simsek et al. 2024).

### Dose–response and synergy analysis

To evaluate the interaction between TMZ and DNF, U87MG and T98G cells were treated with single agents and combinations using a 3 × 3 dose–response matrix generated from half-IC_50_, IC_50_, and 2 × IC_50_concentrations. Cell viability was determined using the MTT assay, and the resulting dose–response data were analyzed using the SynergyFinder platform. Drug interaction effects were evaluated using the Bliss Independence, Highest Single Agent (HSA), Loewe Additivity, and Zero Interaction Potency (ZIP) reference models (Zheng et al. 2022). Synergy scores were interpreted according to established SynergyFinder criteria, where scores > 10 indicate synergistic interactions, scores between − 10 and 10 indicate additive interactions, and scores < − 10 indicate antagonistic interactions (Oylumlu et al. 2026).

### Irradiation setup

To simulate clinical conditions, 5 × 10^3^ cells per well of GBM cells were seeded into 96-well sterile culture plates. Based on the IC_50_values determined for each cell line, TMZ and DNF were administered. In the following experiment, cells were exposed to fractionated irradiation of 2 Gy/day, achieving a cumulative dose of 10 Gy over five following days. Irradiation was performed using the Synergy linear accelerator (ELEKTA) using a 6 MV photon beam (Iorgulescu et al. 2021; Tsai et al. 2021).

### Clonogenic assay

The effects of TMZ and DNF on GBM cell proliferation were analyzed by colony formation assay. U87MG and T98G cells were seeded at 1.5 × 10^3^cells per well in 6-well sterile culture plates and incubated overnight for adhesion. After an incubation period of about 2 weeks, colonies were stained with 0.1% crystal violet (Sigma-Aldrich, #C0775, 100 g) to visualize the cell clusters and quantified to evaluate proliferation (Omoruyi et al. 2020).

### Invasion assay

GBM cells were seeded into 12-well culture plates. Subsequently, cells were exposed to IC_50_ values of TMZ and DNF. After 24 h of administration, cells were harvested and adjusted to a density of 8 × 10^4^cells per well. Cells were seeded into Matrigel-coated inserts and incubated for 48 h (Cheng et al. 2013). Invaded cells were then fixed and stained using the Hema-3 kit, and quantified under a microscope to evaluate treatment effects on cell invasion (Thermo Fisher Scientific, #122–911).

### Migration assay

A wound-healing assay was performed to evaluate the effects of DNF on GBM cell migration. Briefly, 2 × 10^5^cells were seeded into each well of 12-well culture plates (Liu et al. 2021). After scratch formation, cells were treated with TMZ and DNF at their respective IC_50_ concentrations. To minimize the potential contribution of cell proliferation to wound closure, treatments were prepared in culture medium supplemented with 1% FBS. Images of the wound area were acquired at 0, 6, 12, and 24 h using × 4 magnification, and wound closure was quantified to assess cell migration.

#### Immunocytochemistry

Immunofluorescence staining was performed to assess the expression of p-MET (Cell Signaling, #3077, 1/50), p-mTOR (Invitrogen, #PA5-104,898, 1/100), p-ERK (Cell Signaling, #4370, 1/100), p-VEGFR2 (Abcam, #ab38473, 1/50), VEGFR2 (Abcam, #ab2349, 1/100), p-STAT3 (Cell Signaling, #9145, 1/100), STAT3 (Abcam, #AB76315, 1/100), NF-κB p65 (Cell Signaling, #8242, 1/400), and cleaved caspase-3 (cCASP3; Cell Signaling, #9661, 1/200) in GBM cells. Following fixation, permeabilization, and blocking, cells were incubated with primary antibodies overnight at 4 °C, followed by Alexa Fluor 488-conjugated secondary antibody (Pandey et al. 2019; Parker 2016). Subsequent to the incubation period, coverslips were sealed onto slides using Mounting Medium with DAPI (Vector Labs, #H-1200–10) (Wagner et al. 2019; Temizci et al. 2023).

#### Parallel artificial membrane permeability (PAMPA) assay

The permeability of DNF across the BBB was evaluated using the Parallel Artificial Membrane Permeability Assay (PAMPA-BBB; BioAssay Systems, #PMBBB-096) according to the manufacturer’s instructions. Briefly, 300 μL of PBS were added to each well of the acceptor plate, while 5 μL of BBB lipid solution were applied to the donor plate membrane. DNF solutions were prepared and introduced into the donor wells. Permeability control compounds provided in the kit were included to validate assay performance. Following incubation under standard assay conditions according to the manufacturer’s protocol, samples from the acceptor plate were collected and transferred into microcentrifuge tubes. Absorbance spectra were measured using a microplate reader in the range of 200–500 nm at 10 nm intervals. The permeability of DNF was assessed by comparing its absorbance profile with those of permeability control compounds and equilibrium standards.

#### Statistical analysis

Cell viability and protein expression levels were analyzed and statistically compared between experimental groups. All experiments were performed in at least three independent biological replicates (*n* = 3). Statistical analyses were conducted using GraphPad Prism version 10.0 software. For single-time-point comparisons, one-way analysis of variance (ANOVA) followed by Dunnett’s post-hoc test was used. For time-course experiments presented in Fig. 2, two-way ANOVA followed by Dunnett’s multiple comparisons test was performed, with comparisons made between treatment groups within each time point. The data are presented as the mean ± standard deviation (SD), and* p*-values of less than 0.05 were considered statistically significant.

## Results

### *In silico *prediction identified multiple phase I and II metabolites of DNF

The 3D structures of TMZ (PubChem CID: 5394) and DNF (PubChem CID: 25,191,001) were retrieved from the PubChem database (Supplementary Fig. 1). In silico metabolic prediction using GLORYx identified a total of 12 potential metabolites for TMZ and 29 for DNF. Detailed information for each metabolite, including priority scores, predicted biotransformation reactions, and atom counts, is provided in Supplementary Table 1.

### *In silico *analysis suggested potential BBB permeability for several predicted DNF metabolites

The SMILES representations of all predicted metabolites were individually analyzed using the LogBB_Pred server to assess their BBB permeability. Among the predicted metabolites, 25 out of 29 DNF metabolites (86.2%) were classified as BBB-permeable (BBB +), whereas 8 out of 12 TMZ metabolites (66.7%) demonstrated similar permeability (Supplementary Table 1). These findings suggest that certain predicted DNF metabolites may possess relatively higher theoretical BBB permeability potential compared to TMZ metabolites. However, these observations are based solely on computational prediction models and should not be interpreted as direct evidence of CNS penetration or in vivo BBB permeability. Therefore, further experimental and in vivo validation studies are required.

### The effects of TMZ and DNF on U87MG and T98G cell viability

**Fig. 1:**
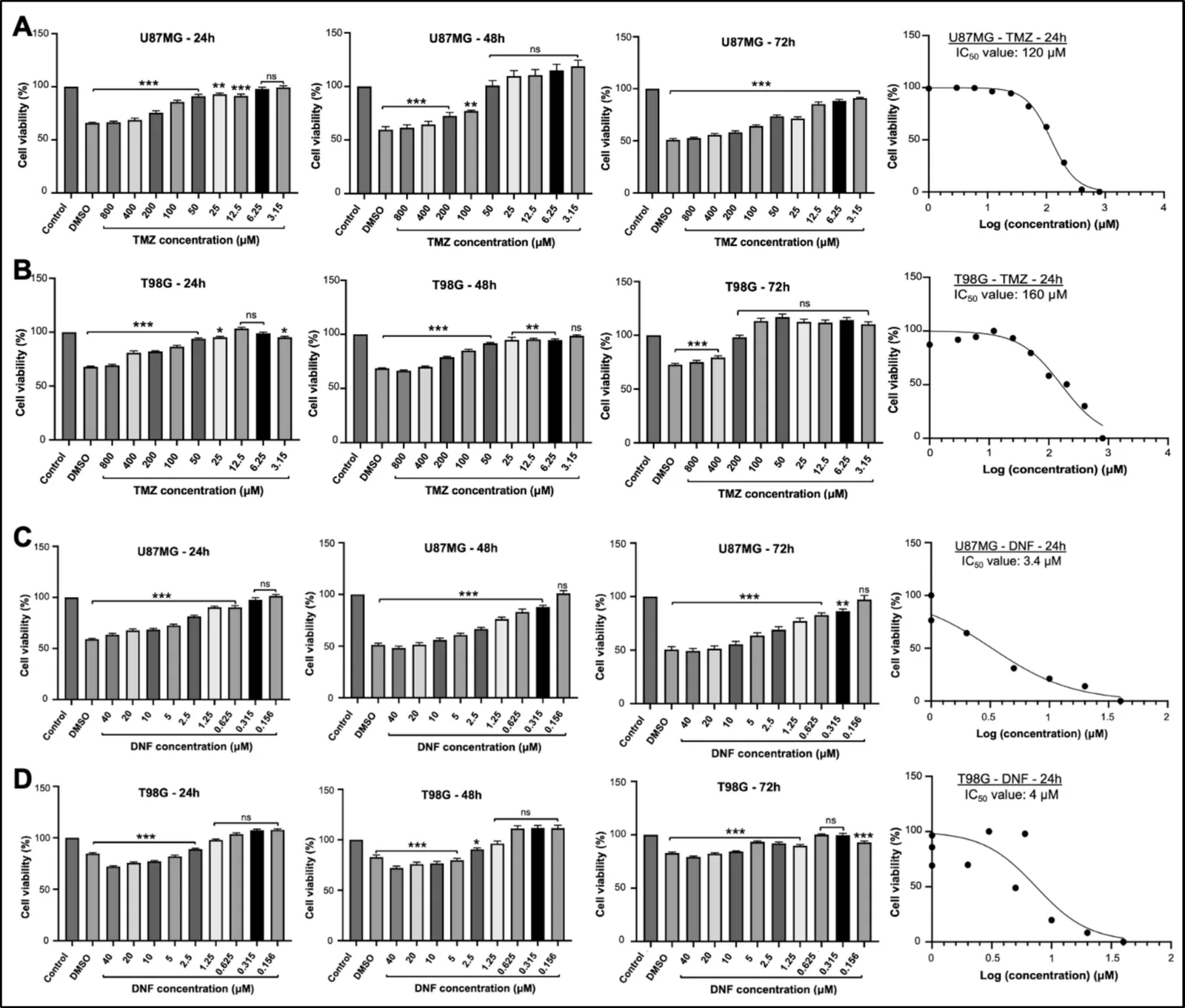
Effect of TMZ and DNF on the viability of U87MG and T98G GBM cells. **A**–**B** TMZ-induced effects on cell viability in U87MG and T98G cells at 24, 48, and 72 h, as assessed by the MTT assay. TMZ reduced cell viability in a dose- and time-dependent manner, with higher IC_50_ values observed in T98G cells, consistent with their relatively lower TMZ sensitivity reported in the literature. **C–D** DNF-induced effects on cell viability in U87MG and T98G cells. DNF significantly decreased cell viability in both cell lines and exhibited markedly lower IC_50_ values compared to TMZ, indicating higher potency. All data are presented as mean ± SD from three independent biological experiments (*n* = 3). Statistical significance was determined using one-way ANOVA followed by Dunnett’s multiple comparison test (ns: *p* > 0.05, *p < 0.05, **p < 0.01, ***p < 0.001) (TMZ, temozolomide; DNF, donafenib; SD, standard deviation)

Dose–response analyses of TMZ and DNF were performed in U87MG and T98G cells across 24, 48, and 72 h. TMZ treatment resulted in a time- and dose-dependent decrease in cell viability in both cell lines. In U87MG cells, the IC_50_ value for TMZ at 24 h was calculated as 120 µM (Fig. 1A). Consistent with the TMZ resistance profile reported in the literature for T98G cells (Keam and Duggan 2021), the IC_50_ value in T98G cells was higher (160 µM), indicating relatively lower sensitivity to TMZ treatment (Fig. 1B).

In contrast, DNF demonstrated markedly higher potency compared to TMZ in both cell lines. In U87MG cells, DNF significantly reduced cell viability across a wide range of concentrations, with a 24 h IC_50_ value of 3.4 µM (Fig. 1C). Similarly, in T98G cells, DNF exhibited strong cytotoxic effects, with an IC_50_ value of 4 µM at 24 h (Fig. 1D). Overall, DNF showed substantially greater anti-proliferative activity than TMZ in both TMZ-sensitive and TMZ-resistant GBM cell lines.

### Synergy analysis of TMZ and DNF combinations in GBM cells

**Fig. 2:**
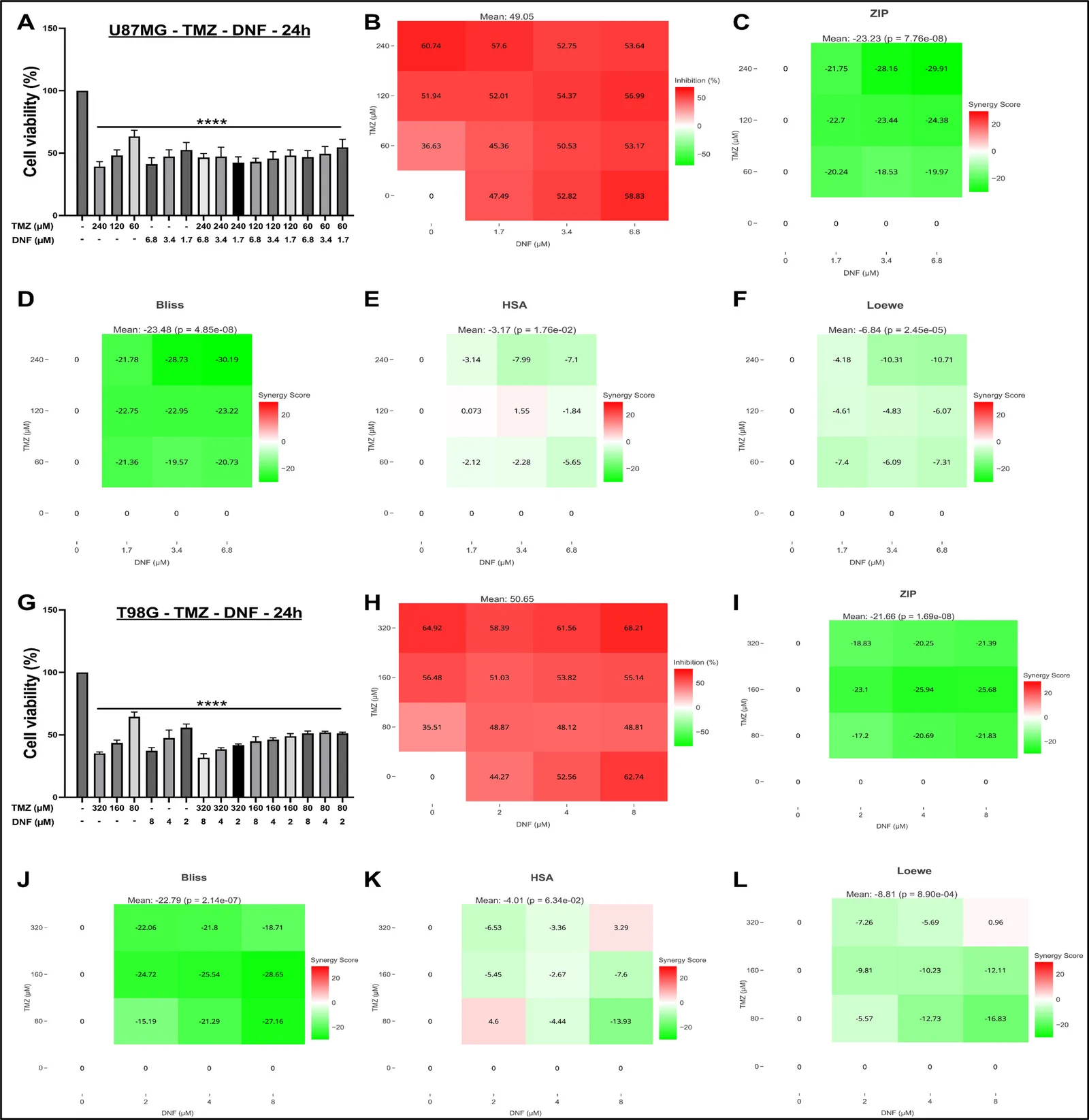
Synergy analysis of TMZ and DNF combinations in U87MG and T98G GBM cells after 24 h treatment. Cell viability of **A** U87MG and **G** T98G cells following treatment with TMZ and DNF alone or in combination, as determined by the MTT assay. Response matrices showing inhibition rates (%) for TMZ–DNF combinations in **B** U87MG and **H** T98G cells. Drug interaction effects were evaluated using the **C, I** ZIP, **D, J** Bliss Independence, **E, K** HSA, and **F, L** Loewe Additivity reference models. Green and red colors indicate antagonistic and synergistic interactions, respectively. Synergy scores were interpreted according to SynergyFinder criteria, where scores > 10 indicate synergistic interactions, scores between − 10 and 10 indicate additive interactions, and scores < − 10 indicate antagonistic interactions. Data are presented as mean ± SD of three independent experiments (*n* = 3). CI values for all tested dose combinations are provided in Supplementary Table 2 (TMZ, temozolomide; DNF, donafenib; SD, standard deviation; ZIP, Zero Interaction Potency; HSA, Highest Single Agent; CI, Combination Index)

To evaluate the interaction between TMZ and DNF, U87MG and T98G cells were treated with single agents and their combinations for 24 h. Combination treatment significantly reduced cell viability compared with the untreated control group in both cell lines (Fig. 2A, G). The SynergyFinder response matrices demonstrated mean inhibition rates of 49.05% and 50.65% in U87MG and T98G cells, respectively (Fig. 2B, H). Synergy analysis revealed predominantly non-synergistic interactions between TMZ and DNF in both cell lines. In U87MG cells, the mean ZIP, Bliss, HSA, and Loewe scores were − 23.23, − 23.48, − 3.17, and − 6.84, respectively (Fig. 2C − F). Similarly, in T98G cells, the corresponding scores were − 21.66, − 22.79, − 4.01, and − 8.81, respectively (Fig. 2I − L). According to the SynergyFinder classification criteria, Bliss and ZIP analyses consistently indicated antagonistic interactions in both cell lines, whereas HSA and Loewe analyses suggested predominantly additive effects. Overall, synergy heatmaps demonstrated that antagonistic regions predominated across most dose combinations in both cell lines. These findings were further supported by CI analysis, which showed CI values > 1 for all tested combinations, indicating the absence of synergistic interactions (Supplementary Table 2).

### Co-administration of DNF with TMZ and radiotherapy potently reduced tumor cell viability in GBM cells

**Fig. 3:**
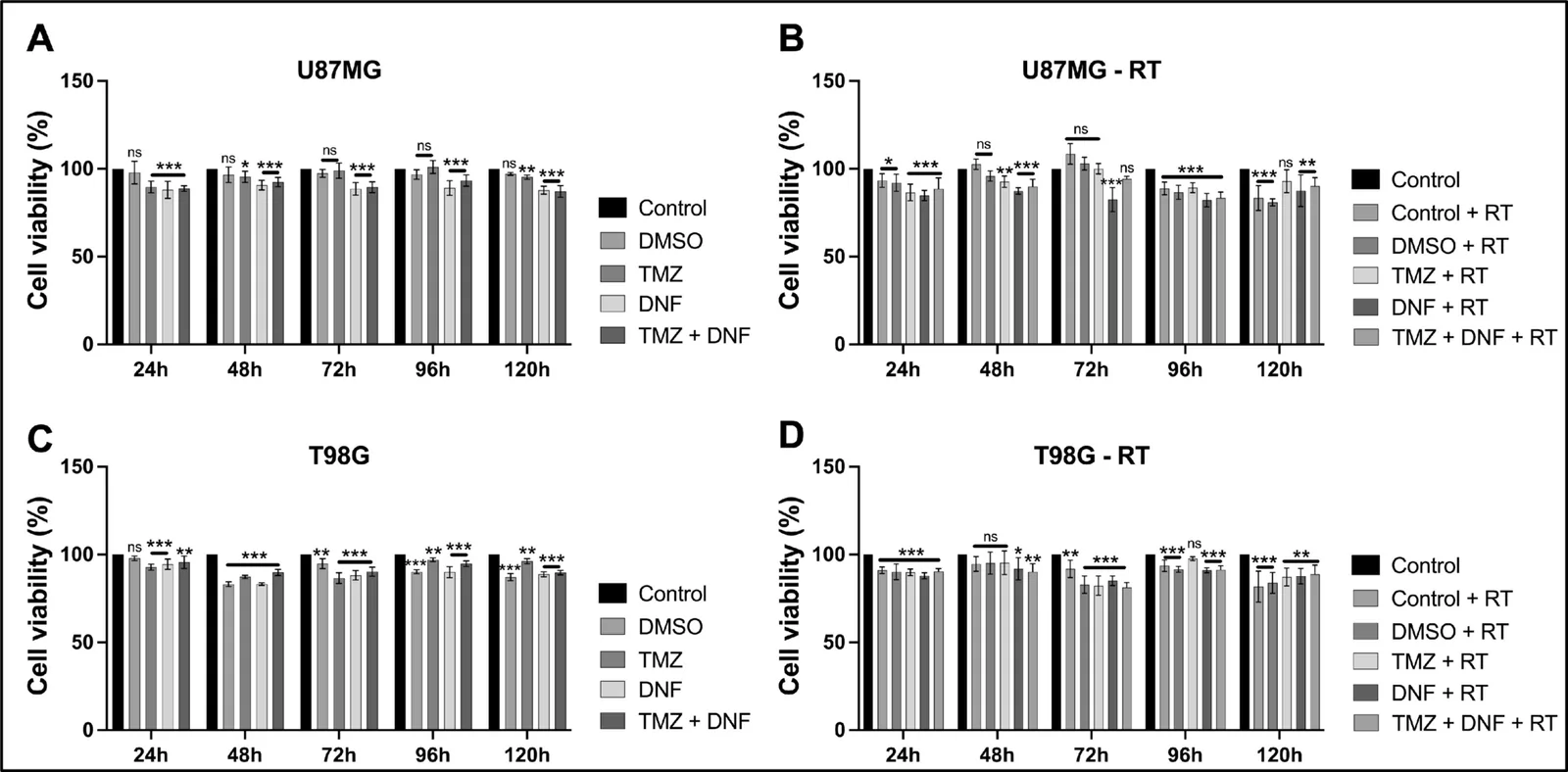
Effects of TMZ, DNF and RT administered individually or in combinations on GBM cell viability over time. **A**–**B** Effects of TMZ and DNF, individually or in combination, on U87MG cell viability at 24, 48, 72, 96, and 120 h, in the presence or absence of RT. DNF-containing treatment regimens were generally associated with lower cell viability values across multiple time points. **C**–**D** Effects of TMZ and DNF on TMZ-resistant T98G cells. DNF-containing combinations, particularly DNF + RT and TMZ + DNF + RT, maintained reduced cell viability across the evaluated time points. Data are presented as mean ± SD of three independent biological experiments (*n* = 3). Statistical significance was determined using two-way ANOVA followed by Dunnett’s multiple comparisons test (ns: *p* > 0.05, *p < 0.05, **p < 0.01, ***p < 0.001) (TMZ, temozolomide; DNF, donafenib; RT, radiotherapy; SD, standard deviation)

Combined treatment with TMZ and DNF resulted in a significant reduction in U87MG cell viability across the evaluated time points. DNF-containing treatments generally demonstrated stronger anti-proliferative effects than TMZ alone, whereas the DMSO vehicle control showed no significant effect on cell viability compared with untreated controls (Fig. 3A).

To better reflect clinical treatment strategies, radiotherapy (RT) was incorporated into the experimental design. In U87MG cells, treatment regimens containing DNF (DNF + RT and TMZ + DNF + RT) were associated with lower viability values across multiple time points, supporting the anti-proliferative activity of DNF-containing combinations in the presence of RT (Fig. 3B).

In the TMZ-resistant T98G cell line, treatment-associated reductions in viability were observed across the evaluated time points. DNF-containing combinations generally maintained lower viability levels, particularly in the RT-treated groups. Notably, DNF + RT and TMZ + DNF + RT groups continued to show reduced viability at later time points, suggesting a potential contribution of DNF to treatment response in TMZ-resistant cells (Fig. 3C, D). No statistically significant reduction in viability was observed in DMSO-treated groups at the primary 24 h experimental time point compared with untreated controls, suggesting that DMSO-related cytotoxicity did not substantially influence the principal findings (Fig. 3A, C).

### DNF suppresses colony formation, invasion, and migration in GBM cells

**Fig. 4:**
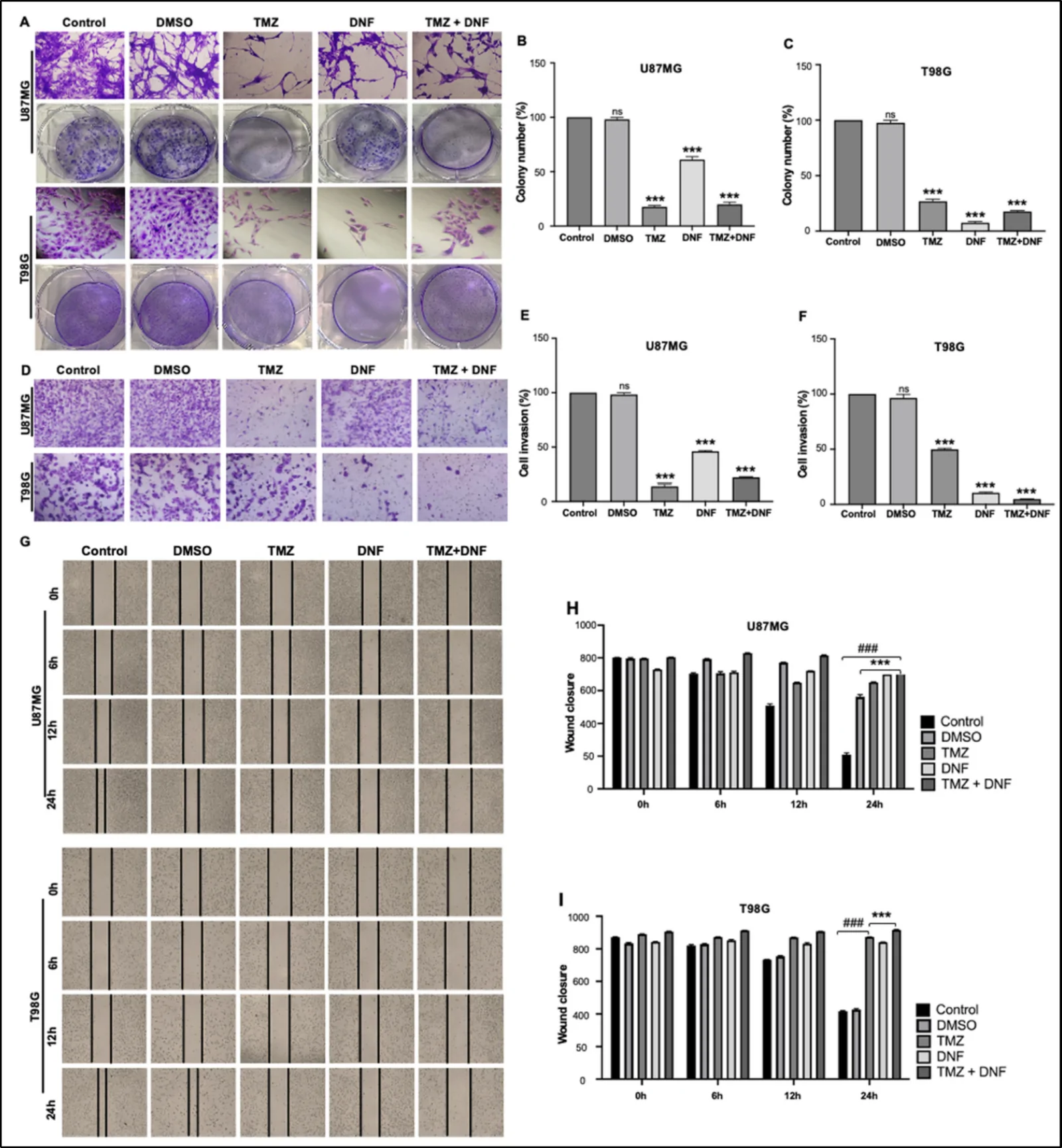
Effects of TMZ and DNF on colony formation, invasion, and migration of GBM cells. **A**–**C** Representative images and quantitative analysis of colony formation in U87MG and T98G cells following TMZ and DNF treatment. Both agents reduced colony formation compared to control, with differential responses observed between cell lines. **D**–**F** Effects of TMZ and DNF on cell invasion. Both treatments significantly decreased the invasive capacity of GBM cells, with cell line–dependent differences in response. **G**–**I** Wound healing assay showing the effects of TMZ and DNF on cell migration. Both treatments reduced wound closure over time, with comparable effects observed between treatment groups. Data are presented as mean ± SD from three independent biological experiments (*n* = 3). Statistical significance was determined using one-way ANOVA followed by Tukey’s post hoc test. Symbols (#) indicate comparisons within the same treatment group relative to its baseline (0 h), whereas symbols (*) indicate comparisons between treatment groups and control at the same time point (ns:* p* > 0.05, ^###^*p* < 0.001, ***p < 0.001) (TMZ, temozolomide; DNF, donafenib; SD, standard deviation)

The effects of TMZ and DNF on the clonogenic capacity of GBM cells were assessed using colony formation assays. Both TMZ and DNF reduced colony numbers compared to the control group; however, their effects differed between cell lines. In U87MG cells, TMZ exhibited a stronger inhibitory effect on colony formation, whereas in T98G cells, DNF treatment resulted in a more pronounced reduction (Fig. 4A–C).

Cell invasion assays demonstrated that both TMZ and DNF reduced the invasive capacity of GBM cells; however, the extent of this effect varied between cell lines. In U87MG cells, TMZ treatment resulted in a more pronounced reduction in invasion compared to DNF. In contrast, in TMZ-resistant T98G cells, DNF exhibited a stronger inhibitory effect than TMZ. Notably, combination treatment (TMZ + DNF) produced the greatest reduction in invasion in T98G cells. No significant differences were observed between the DMSO-treated and control groups (Fig. 4D–F).

The migratory potential of GBM cells was evaluated using a wound healing assay. Both TMZ and DNF significantly reduced cell migration compared to control groups at corresponding time points. In addition, a time-dependent decrease in wound closure was observed within treatment groups (Fig. 4G–I).

### DNF treatment modulates proliferation- and apoptosis-related marker expression in GBM cells

**Fig. 5:**
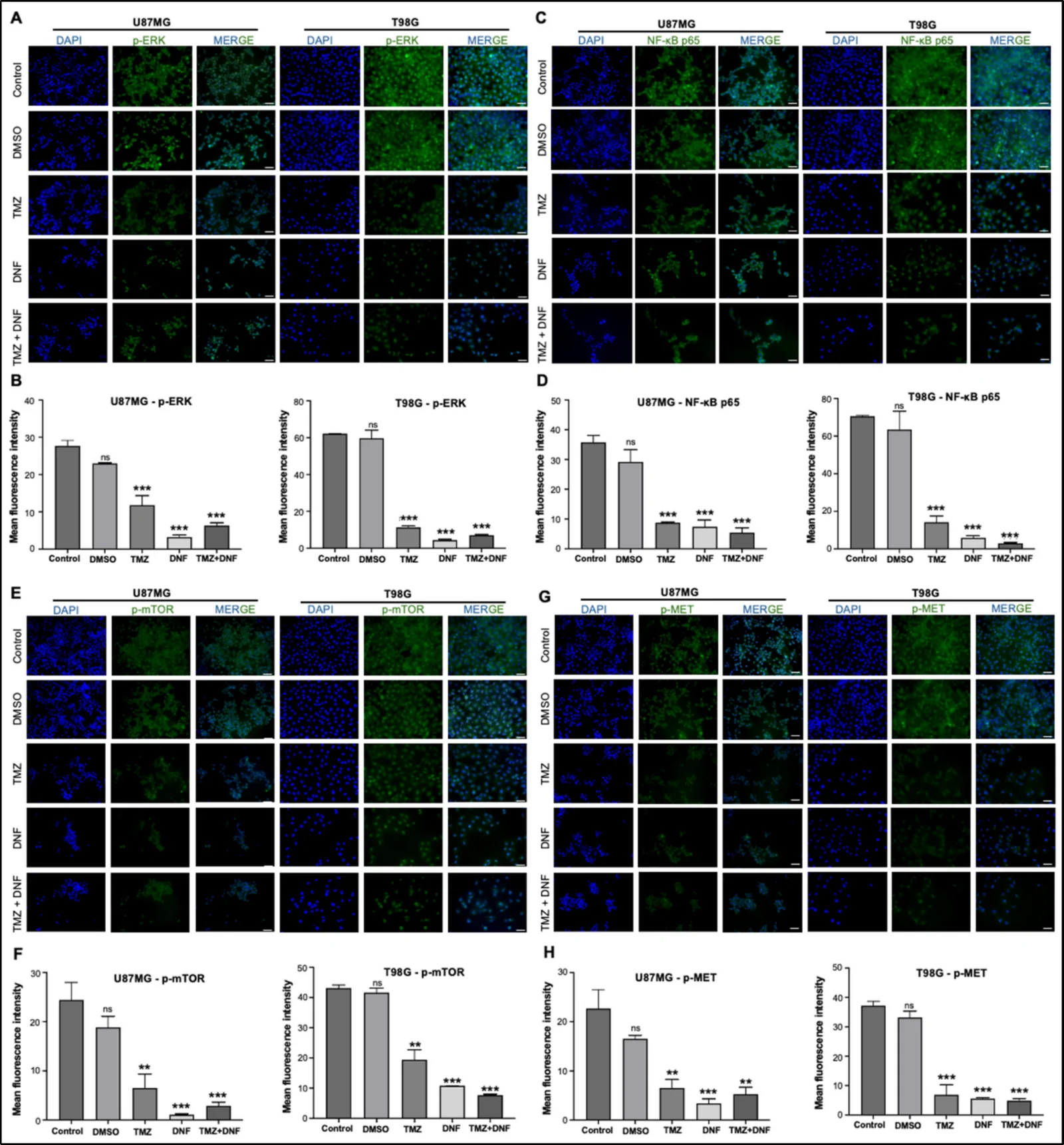

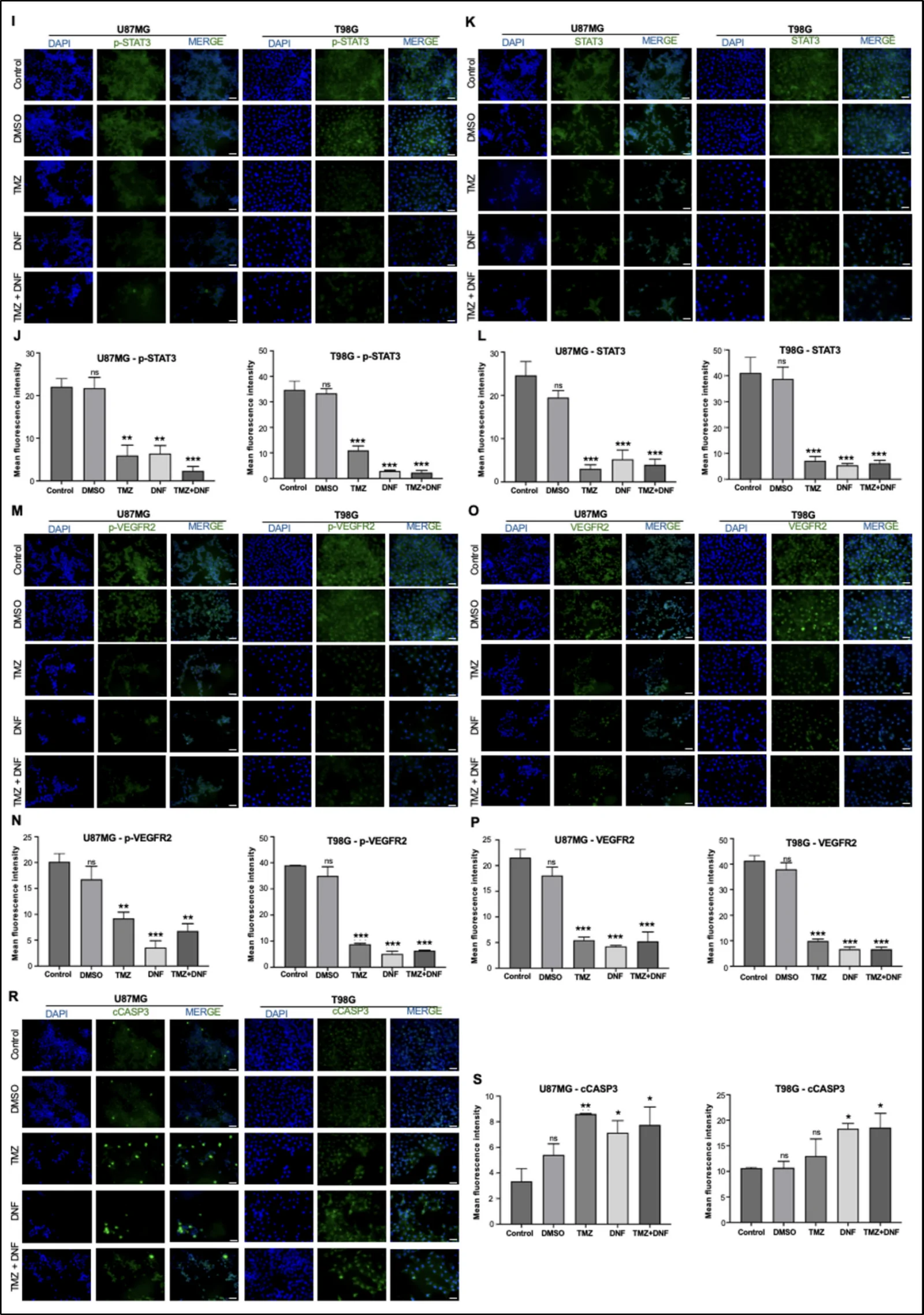
Immunocytochemical analysis of proliferation- and apoptosis-related marker expression in GBM cells following TMZ and DNF treatment. **A**–**D** Representative immunofluorescence images and quantification of p-ERK and NF-κB p65 expression in U87MG and T98G cells. Reduced immunofluorescence signals for both markers were observed following TMZ and DNF treatment, with DNF-containing groups generally exhibiting lower signal intensity compared with TMZ-treated cells. **E**–**H** Immunofluorescence analysis of p-mTOR and p-MET expression. Both markers exhibited reduced immunoreactivity following treatment, particularly in DNF-containing groups. **I**–**L** Expression of p-STAT3 and total STAT3. Reduced immunofluorescence signals were observed across treatment groups, indicating treatment-associated alterations in markers involved in cell survival and proliferation. **M**–**P** Analysis of p-VEGFR2 and total VEGFR2 expression. Reduced immunoreactivity was observed following TMZ and DNF treatment. **R**–**S** Immunofluorescence analysis of cCASP3. Increased cCASP3 immunoreactivity was observed following treatment, particularly in DNF-treated groups and TMZ-resistant T98G cells. Cells were stained with DAPI (nuclei, blue) and target proteins (green). Scale bar: 100 μm. Data are presented as mean ± SD from three independent biological experiments (*n* = 3). Statistical significance was determined using one-way ANOVA followed by Tukey’s post-hoc test (ns: *p* > 0.05, *p < 0.05, **p < 0.01, ***p < 0.001) (TMZ, temozolomide; DNF, donafenib; cCASP3, Cleaved caspase-3; SD, standard deviation)

Immunocytochemical analysis demonstrated that both TMZ and DNF were associated with reduced expression of several proliferation-related signaling markers in GBM cells. Notably, DNF treatment generally resulted in lower p-ERK and NF-κB p65 immunofluorescence signals compared with TMZ-treated cells, as reflected by reduced fluorescence intensity and cell density (Fig. 5A–D).

Similarly, p-mTOR and p-MET expression levels were decreased following both individual and combined treatments, with DNF-containing groups showing consistent inhibitory effects. These changes were not attributable to the vehicle (Fig. 5E–H).

In addition, reduced p-STAT3 and total STAT3 immunofluorescence signals were observed across treatment groups, indicating treatment-associated alterations in markers involved in cell survival and proliferation (Fig. 5I–L).

Likewise, p-VEGFR2 and total VEGFR2 immunoreactivity appeared reduced following TMZ and DNF treatment, suggesting modulation of VEGFR2-associated signaling markers in GBM cells (Fig. 5M–P).

Analysis of the apoptotic marker cCASP3 demonstrated increased immunofluorescence signals following treatment. In U87MG cells, both TMZ and DNF increased cCASP3 expression, whereas in TMZ-resistant T98G cells, DNF, either alone or in combination with TMZ, was associated with a more pronounced increase in cCASP3 immunoreactivity (Fig. 5R–S).

### Permeability of TMZ and DNF across the BBB Assessed by the PAMPA assay

**Fig. 6:**
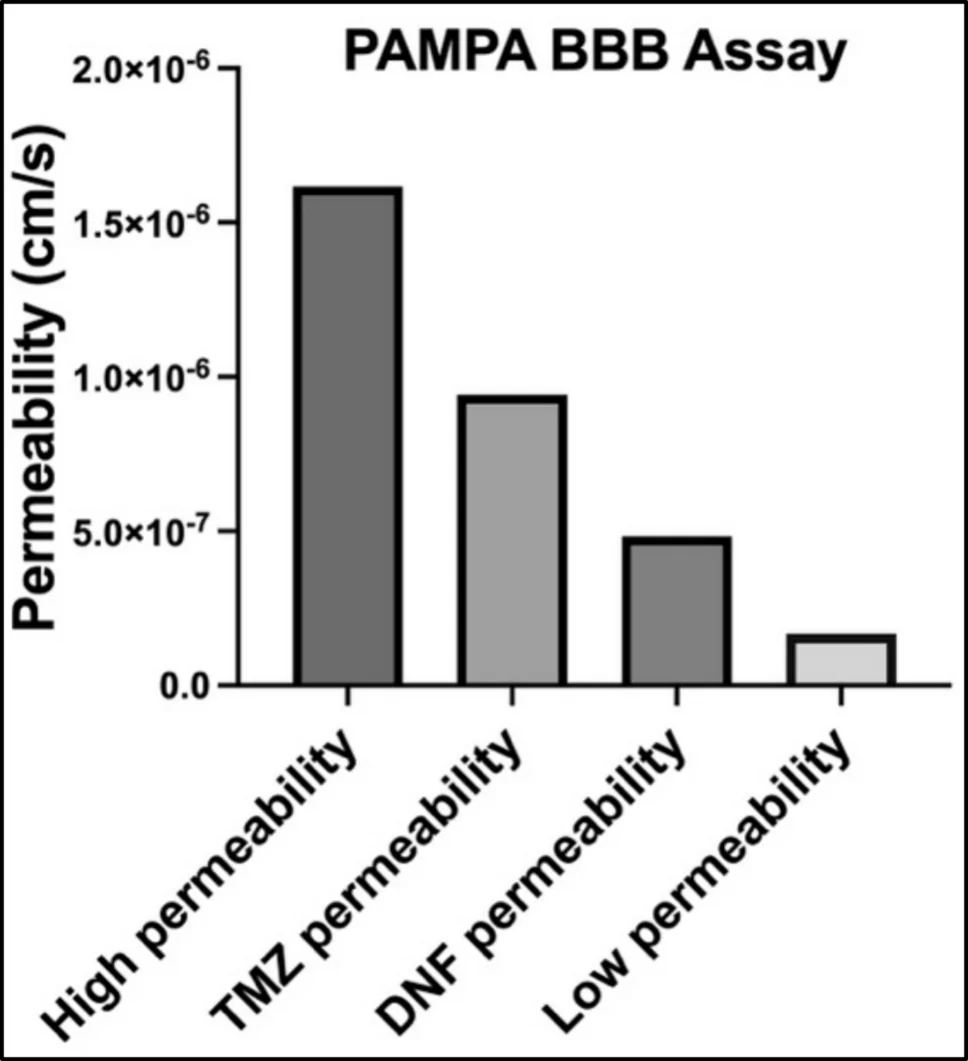
The graphs illustrating the BBB permeability of TMZ and DNF inhibitors assessed using the PAMPA kit. The data indicate that both TMZ and DNF exhibit permeability across the BBB, with TMZ showing higher permeability compared to DNF. Both compounds demonstrated greater permeability than the low-permeability reference (TMZ, temozolomide; DNF, donafenib; PAMPA, Parallel Artificial Membrane Permeability Assay; BBB, blood–brain barrier)

The PAMPA-BBB assay demonstrated that TMZ exhibited higher permeability across the BBB compared to DNF, whereas DNF showed comparatively lower permeability under the same experimental conditions (Fig. 6). These findings indicate limited passive BBB permeability of the parent DNF compound under the experimental conditions used in the PAMPA-BBB model. Importantly, these results reflect the permeability characteristics of the parent compounds and should not be directly compared with the in silico BBB permeability predictions generated for the predicted metabolites.

## Discussion

DNF, a novel small-molecule multikinase inhibitor targeting VEGFR and PDGFR signaling pathways, has demonstrated potent antitumor activity in various cancer models (Niu et al. 2021). However, its effects in GBM have not been extensively investigated. In this study, we provide evidence that DNF significantly inhibits proliferation and migration in both TMZ-sensitive and TMZ-resistant GBM cell lines.

The IC_50_values determined for TMZ in our study were consistent with previous reports (Lee 2016), supporting the reliability of our experimental model. Notably, DNF treatment resulted in significant reductions in cell proliferation in both U87MG and T98G cells, either as a monotherapy or in combination with TMZ and RT.

Although combination treatment with TMZ further reduced cell viability in both U87MG and T98G cells, SynergyFinder analyses revealed predominantly non-synergistic interactions, with Bliss and ZIP models indicating antagonistic effects and HSA and Loewe models suggesting mainly additive interactions. These findings suggest that the antitumor activity observed in the combination groups may largely reflect the individual effects of each agent rather than a true synergistic interaction. Notably, a similar interaction profile was observed in both TMZ-sensitive and TMZ-resistant cell lines, indicating that the lack of synergy was not solely dependent on TMZ responsiveness.

Previous studies have shown that TMZ reduces colony formation more effectively in TMZ-sensitive GBM cells than in resistant models such as T98G (de Souza et al. 2022). Consistent with these findings, our results are in agreement with the existing literature, demonstrating that TMZ treatment induces a more significant reduction in colony formation in sensitive cells compared to resistant cells.

Combination strategies have been reported to enhance antitumor effects in GBM models. For instance, co-treatment approaches have demonstrated improved antiproliferative effects compared to monotherapies (Pandey et al. 2019). Despite the absence of synergistic interactions, DNF retained substantial antiproliferative activity in both cell lines. Furthermore, studies in other cancer types have demonstrated that DNF exerts strong anti-proliferative effects compared to related inhibitors (Xiong et al. 2024), supporting its potential relevance for further investigation in GBM.

Although GBM rarely metastasizes, its highly infiltrative nature contributes to local recurrence and poor clinical outcomes (Demuth and Berens 2004). In this context, targeting invasion and migration is of critical importance. Previous studies have demonstrated that DNF inhibits invasion in other cancer models (Liang et al. 2025), and TMZ is also known to reduce GBM cell invasion. Consistent with these findings, our results show that DNF significantly suppresses invasion in GBM cells. Similarly, TMZ has been widely reported to inhibit GBM cell migration (Pekmez and Kılcı 2022), while DNF has been shown to reduce migration in a dose-dependent manner in other tumor models (Liang et al. 2025). In agreement with the literature, our findings demonstrate that both TMZ and DNF impair migratory capacity in GBM cells, as evidenced by reduced wound closure compared to control groups.

Several multikinase inhibitors, including sorafenib (Khot et al. 2025), regorafenib (Tünbekici et al. 2025), lenvatinib (Rha et al. 2025), and cabozantinib (Wen et al. 2018), have previously been investigated as potential therapeutic agents for GBM. Although these agents target signaling pathways involved in tumor growth, angiogenesis, and invasion (Salbini et al. 2025), their overall clinical efficacy has remained limited due to challenges such as poor BBB penetration, intratumoral heterogeneity, and the development of treatment resistance (Biwash “Advancing Personalized Cancer Care: Integrating CRISPR/Cas9 with Next-Generation Sequencing Technologies” 2024; Parker et al. 2016). Therefore, the identification of novel multitargeted therapeutic approaches remains an important objective in GBM research. In this context, the present study provides evidence regarding the antiproliferative, antimigratory, and apoptosis-associated effects of DNF in both TMZ-sensitive and TMZ-resistant GBM models, supporting its potential for further investigation as a multitargeted therapeutic candidate.

Sorafenib, a RAF inhibitor approved by the FDA, served as a precursor for the development of DNF, which was designed to improve pharmacological stability and reduce toxicity (Liu et al. 2022; Li et al. 2020). Previous studies have demonstrated that DNF effectively suppresses ERK1/2 phosphorylation, with greater inhibitory potency compared to related agents (Xiong et al. 2024). Consistent with these findings, our results show that DNF markedly reduces p-ERK levels in GBM cells, including TMZ-resistant T98G cells.

NF-κB signaling is another key regulator of tumor survival and proliferation. Previous reports have shown that TMZ treatment decreases NF-κB p65 expression in GBM cells (Avci et al. 2020). In agreement with this, our findings demonstrate that both TMZ and DNF reduce NF-κB p65 levels, supporting treatment-associated changes in markers related to pro-survival signaling pathways.

The mTOR pathway plays a central role in regulating cell growth, proliferation, and survival (Rascio et al. 2021). Consistent with previous findings demonstrating that TMZ inhibits p-mTOR expression in GBM cells (Zhou et al. 2017), our study showed reduced p-mTOR immunoreactivity following TMZ and DNF treatment. Similarly, p-MET, a key driver of tumor progression, was reduced following treatment, in line with previous reports (Gallo et al. 2024; Vengoji et al. 2019).

STAT3 is a critical transcription factor involved in tumor cell survival and inflammation, and its phosphorylated form (p-STAT3) is frequently associated with oncogenic signaling (Samad et al. 2025). Previous studies have reported that TMZ inhibits p-STAT3 expression in GBM cells (Liu et al. 2019). Consistent with these findings, our results indicate that both STAT3 and p-STAT3 levels are reduced following TMZ and DNF treatment.

GBM is a highly vascularized tumor that promotes angiogenesis through VEGFR2 signaling (Wang et al. 2022). In agreement with studies showing that DNF inhibits VEGFR2 expression in other cell types (Xiong et al. 2024), our findings reveal a reduction in both VEGFR2 and p-VEGFR2 levels following treatment, suggesting treatment-associated changes in markers related to angiogenic signaling.

Apoptosis induction is a key mechanism of anti-cancer therapy. Previous studies have shown that TMZ increases cCASP3 levels in tumor cells (Das et al. 2004; Xiao et al. 2013), while DNF has also been reported to promote apoptosis in cancer models (Liang et al. 2025). Notably, in our study, TMZ alone did not significantly increase cCASP3 levels in TMZ-resistant T98G cells, whereas DNF, both alone and in combination with TMZ, was associated with increased cCASP3 immunoreactivity in TMZ-resistant T98G cells.

**Fig. 7:**
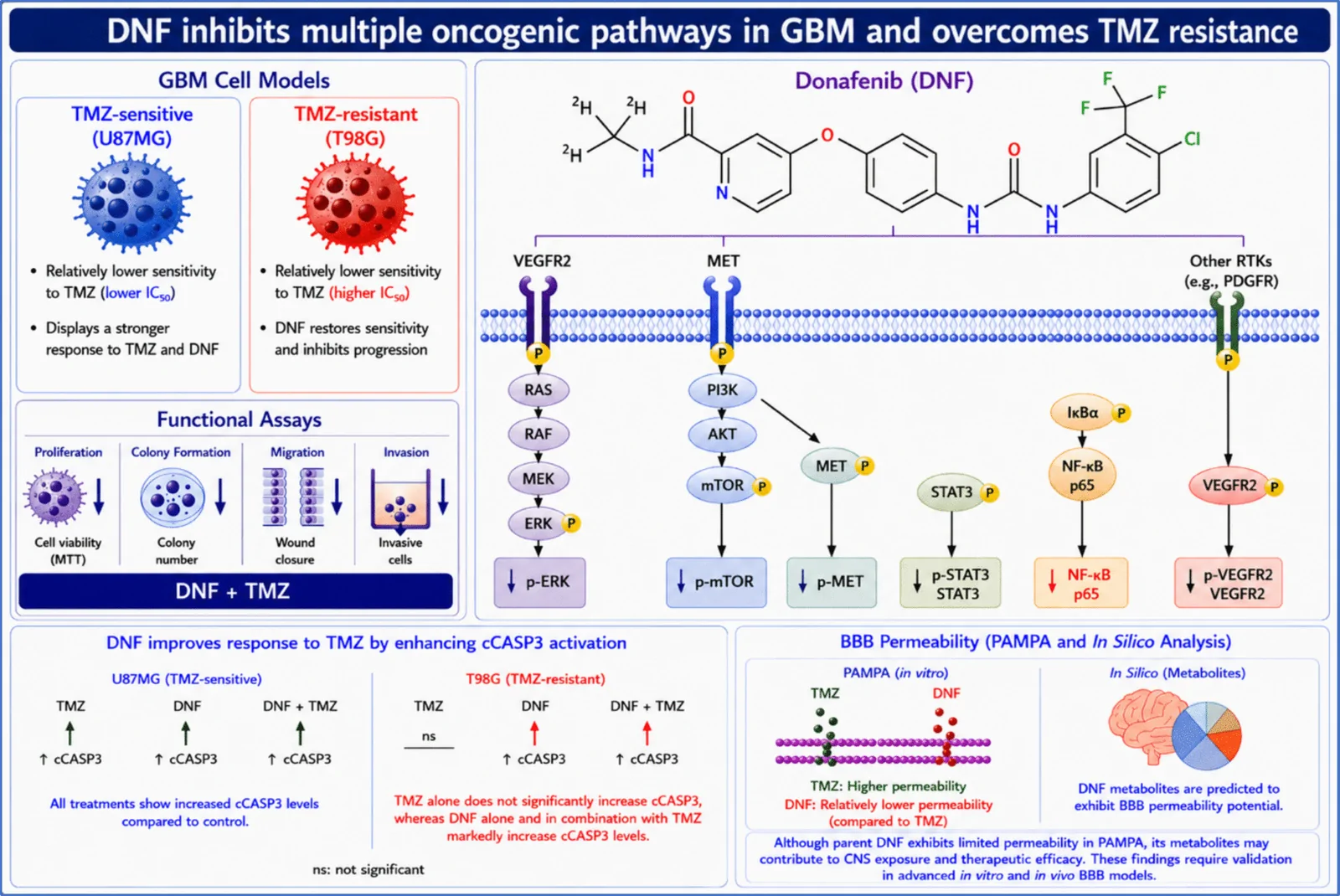
Proposed mechanism of DNF-mediated suppression of oncogenic signaling and reversal of TMZ resistance in GBM. DNF inhibits multiple receptor tyrosine kinase–mediated signaling pathways, including VEGFR2 and MET, leading to the downregulation of key downstream effectors such as ERK, mTOR, STAT3, NF-κB p65, and VEGFR2. This results in reduced proliferation, migration, invasion, and colony formation in both U87MG and T98G cells. In addition, DNF treatment was associated with increased cCASP3 expression, particularly in T98G cells. BBB analysis demonstrated higher permeability of TMZ compared to DNF in the PAMPA model, whereas in silico predictions suggested potential BBB permeability for certain predicted DNF metabolites. These observations require further experimental and in vivo validation (DNF, donafenib; TMZ, temozolomide; GBM, Glioblastoma; BBB, blood–brain barrier; PAMPA, Parallel Artificial Membrane Permeability Assay)

Overall, these findings suggest that DNF treatment is associated with changes in multiple signaling-related markers, including ERK, STAT3, NF-κB, MET, and VEGFR2. Given the extensive crosstalk between these pathways, particularly the regulation of VEGFR2 expression by STAT3 and NF-κB, the observed treatment-associated changes in these markers may contribute to the antitumor effects of DNF in GBM models. A schematic representation summarizing the proposed multi-targeted mechanism of DNF in GBM is provided in Fig. 7.

In this study, in silico analyses suggested that several predicted DNF metabolites may possess theoretical BBB permeability potential, whereas PAMPA-BBB results indicated lower permeability of the parent DNF compound compared with TMZ. However, these findings are not directly contradictory, as the computational analyses evaluated predicted metabolites, whereas the PAMPA-BBB assay assessed the permeability characteristics of the parent compounds. Nevertheless, both in silico predictions and PAMPA-BBB assays have inherent methodological limitations. Therefore, the BBB penetration capacity of DNF and its metabolites requires further validation using advanced in vitro BBB models, pharmacokinetic analyses, and in vivo studies. Importantly, limited BBB permeability of a parent compound does not necessarily preclude therapeutic relevance in GBM. However, the potential contribution of DNF metabolites to CNS exposure and therapeutic activity remains speculative and should be interpreted cautiously until experimentally validated.

Taken together, these findings support further investigation of DNF as a potential multi-targeted therapeutic candidate for GBM, including relatively TMZ-resistant models. However, further mechanistic and pharmacokinetic investigations, particularly in orthotopic GBM animal models, will be necessary to validate its BBB penetration, in vivo therapeutic efficacy, and translational relevance.

## Supplementary Information

Below is the link to the electronic supplementary material.

ESM 1 (PNG 231 KB)

ESM 2 (XLSX 42.4 KB) 

ESM 3 (DOCX 21.4 KB) 

## Data Availability

The data that support the findings of this study are available from the corresponding author, upon reasonable request.

## Abbreviations

*GBM*: Glioblastoma
*WHO*: World Health Organization
*DNF*: Donafenib
*TMZ*: Temozolomide
*BBB*: Blood-brain-barrier
*Gy*: Gray
*VEGFR*: Vascular endothelial growth factor receptor
*PDGFR*: Platelet-derived growth factor receptor
*3D*: The three-dimensional
*SMILES*: Simplified Molecular Input Line Entry System
*LightGBM*: Light Gradient Boosting Machine
*GBDT*: Gradient Boosting Decision Trees
*DMSO*: Dimethyl sulfoxide
*DMEM*: Dulbecco’s Modified Eagle Medium
*FBS*: Fetal bovine serum
*P/S*: Penicillin-streptomycin
*MTT*: 3-(4,5-Dimethylthiazol-2-yl)-2,5-Diphenyltetrazolium Bromide
*ZIP*: Zero Interaction Potency
*HSA*: Highest Single Agent
*CI*: Combination Index
*PBS*: Phosphate-buffered saline
*PFA*: Paraformaldehyde
*BSA*: Bovine serum albumin
*PAMPA*: Parallel Artificial Membrane Permeability Assay
*ANOVA*: One-way analysis of variance
*SDF*: Structure-data files
*RT*: Radiotherapy
*HCC*: Hepatocellular carcinoma
*SD*: Standard deviation.
