# Supplementary material for: Donafenib suppresses glioblastoma progression: potential enhancement of therapeutic response in TMZ-resistant models
Source: Naunyn Schmiedebergs Arch Pharmacol. 2026 Jul 6;399(13):20505–20. doi: 10.1007/s00210-026-05631-z (PMC13695675; doi:10.1007/s00210-026-05631-z)
Supplement: Supplementary file 1 [file 210_2026_5631_MOESM3_ESM.docx]

**Supplementary Table 2.** Combination index (CI) values for TMZ and DNF combinations in **(A)** U87MG and **(B)** T98G cells after 24 h treatment.

A) U87MG cell

| **TMZ (µM)** | **DNF (µM)** | **Cell viability (%)** | **CI value** | **Interpretation** |
| --- | --- | --- | --- | --- |
| 60 | 6.8 | 53.17 | 2.39 | Antagonistic |
| 60 | 3.4 | 50.53 | 1.84 | Antagonistic |
| 60 | 1.7 | 45.36 | 1.92 | Antagonistic |
| 120 | 6.8 | 56.99 | 1.90 | Antagonistic |
| 120 | 3.4 | 54.37 | 1.63 | Antagonistic |
| 120 | 1.7 | 52.01 | 1.45 | Antagonistic |
| 240 | 6.8 | 53.64 | 3.48 | Antagonistic |
| 240 | 3.4 | 52.75 | 2.73 | Antagonistic |
| 240 | 1.7 | 57.60 | 1.57 | Antagonistic |

B) T98G cell

| **TMZ (µM)** | **DNF (µM)** | **Cell viability (%)** | **CI value** | **Interpretation** |
| --- | --- | --- | --- | --- |
| 80 | 8 | 48.81 | 3.37 | Antagonistic |
| 80 | 4 | 48.12 | 2.08 | Antagonistic |
| 80 | 2 | 48.87 | 1.28 | Antagonistic |
| 160 | 8 | 55.14 | 2.63 | Antagonistic |
| 160 | 4 | 53.82 | 1.90 | Antagonistic |
| 160 | 2 | 51.03 | 1.66 | Antagonistic |
| 320 | 8 | 68.21 | 1.57 | Antagonistic |
| 320 | 4 | 61.56 | 1.85 | Antagonistic |
| 320 | 2 | 58.39 | 1.87 | Antagonistic |
